# Dual-Function Antimicrobial Peptides as a Prospective Strategy Against Peri-Implantitis: Bridging Cutaneous Wound Healing and the Peri-Implant Soft-Tissue Seal

**DOI:** 10.3390/medicina62081463

**Published:** 2026-07-28

**Authors:** Laura Maghiar, Andrada Iftode, Andreea-Adriana Neamțu, Teodor-Andrei Maghiar, Andreea Maria Cristea, Cristina Dumitrescu, Alina Anton, Andreea-Mihaela Kis, Valentin-Cristian Iovin, Marge Cristian, Gabriel Armencea, Ruxandra Florina Bodog, Cristina-Adriana Dehelean, Carmen Neamțu, Andrei Paul Tent

**Affiliations:** 1 Department of Psycho-Neurosciences and Rehabilitation, Faculty of Medicine and Pharmacy, University of Oradea, Universității Str., No. 1, 410087 Oradea, Romania; laura.maghiar@uoradea.ro; 2 Department of Dermatovenerology, Clinical County Emergency Hospital Bihor, Gheorghe Doja Str., No. 65, 410169 Oradea, Romania; 3 Department of Toxicology, “Victor Babeș” University of Medicine and Pharmacy of Timisoara, Eftimie Murgu Square, No. 2, 300041 Timișoara, Romania; andradaiftode@umft.ro (A.I.); andreea.neamtu@umft.ro (A.-A.N.); andreea.cristea@umft.ro (A.M.C.); cristina.grosu@umft.ro (C.D.); dolghi.alina@umft.ro (A.A.); cadehelean@umft.ro (C.-A.D.); 4 Research Centre for Pharmaco-Toxicological Evaluation, “Victor Babeș” University of Medicine and Pharmacy of Timisoara, Eftimie Murgu Square, No. 2, 300041 Timișoara, Romania; 5 Department of Pathology, Clinical County Emergency Hospital of Arad, Andrenyi Karoly Str., No. 2–4, 310037 Arad, Romania; 6 Department of Pathology, “Pius Brînzeu” Clinical County Emergency Hospital Timișoara, Liviu Rebreanu Boulevard, No. 156, 300723 Timișoara, Romania; 7 Department of Surgery, Faculty of Medicine and Pharmacy, University of Oradea, Universității Str., No. 1, 410087 Oradea, Romania; 8 Department of Surgery, Pelican Hospital, Corneliu Coposu Str., No. 2, 410450 Oradea, Romania; 9 Department of Management and Communication in Dental Medicine, “Victor Babeș” University of Medicine and Pharmacy of Timisoara, Eftimie Murgu Square, No. 2, 300041 Timișoara, Romania; 10 Doctoral School Department, “Victor Babeș” University of Medicine and Pharmacy of Timisoara, Eftimie Murgu Square, No. 2, 300041 Timișoara, Romania; valentin.iovin@umft.ro; 11 Department III Functional Sciences, Physiology Discipline, “Victor Babeș” University of Medicine and Pharmacy of Timisoara, Eftimie Murgu Square, No. 2, 300041 Timișoara, Romania; 12 Centre of Immuno-Physiology and Biotechnologies (CIFBIOTEH), Department of Functional Sciences, Physiology, “Victor Babeș” University of Medicine and Pharmacy of Timisoara, Eftimie Murgu Square, No. 2, 300041 Timișoara, Romania; 13 Department of Neuroscience, Faculty of Medicine and Pharmacy, University of Oradea, Universității Str., No. 1, 410087 Oradea, Romania; marge.cristian@yahoo.ro; 14 Department of Oral and Maxillofacial Surgery and Radiology, Faculty of Dental Medicine, “Iuliu Hațieganu” University of Medicine and Pharmacy, Victor Babeș Str., No. 8, 400012 Cluj-Napoca, Romania; garmencea@gmail.com; 15 Department of Surgical Disciplines, Faculty of Medicine and Pharmacy, University of Oradea, 1st December Square, No. 10, 410073 Oradea, Romania; bodog.ruxandraflorina@student.uoradea.ro; 16 Doctoral School of Biomedical Sciences, Faculty of Medicine and Pharmacy, University of Oradea, Universității Street, No. 1, 410087 Oradea, Romania; 17 Faculty of Dentistry, “Vasile Goldis” Western University of Arad, Liviu Rebreanu Str., No. 86, 310045 Arad, Romania; neamtu.carmen@uvvg.ro; 18 Department of Surgery I, Clinical County Emergency Hospital of Arad, Andrenyi Karoly Str., No. 2–4, 310037 Arad, Romania; 19 Department of Oral and Maxillo-Facial Surgery, Faculty of Medicine and Pharmacy, University of Oradea, Universității Str., No. 1, 410087 Oradea, Romania; tent_andrei@yahoo.com

**Keywords:** peri-implantitis, antimicrobial peptides, host-defense peptides, LL-37, β-defensins, dental implant coating, biofilm, soft-tissue seal, osseointegration, wound healing, transmucosal seal, peri-implant abutment, zirconia

## Abstract

Peri-implantitis, a biofilm-driven inflammatory disease that causes progressive loss of the bone supporting dental implants, is common and difficult to treat: mechanical debridement cannot fully decontaminate the implant surface, and antibiotic adjuncts act non-selectively while promoting resistance. Antimicrobial peptides (AMPs) have emerged as a promising preventive strategy. As cationic, membrane-disrupting molecules they kill a broad spectrum of organisms with a low propensity to select for resistance, and as host-defense peptides they additionally modulate inflammation and promote epithelial and connective-tissue repair. This review examines AMP-functionalized titanium as a prospective strategy against peri-implantitis through a dermatological lens, drawing on the established roles of the cathelicidin LL-37 and the β-defensins in cutaneous and oral wound healing. We argue that the peri-implant transmucosal interface behaves as a healing epithelial barrier, so that a single class of host-defense peptides can address two goals usually pursued separately—suppressing the peri-implant biofilm and reinforcing the soft-tissue seal. Because peri-implant disease initiates at the transmucosal region, we give particular attention to the abutment or transmucosal collar as the primary sealing target, and we consider how the concept extends to zirconia and hybrid components. The evidence assembled here, however, is predominantly preclinical, derived from in vitro and animal studies, and does not yet demonstrate clinical prevention of peri-implantitis in patients. After surveying peri-implant epidemiology, microbiology, AMP biology, surface-engineering strategies, and the in vivo evidence, we appraise the translational barriers—stability, cytotoxicity, cost, regulation, and the absence of human trials—that remain. We conclude that biologically intelligent, multifunctional peptide coatings represent a rational direction for next-generation implant surfaces.

## 1. Introduction

Endosseous dental implants have become a routine and dependable option for rehabilitating partial and complete edentulism, yet their long-term success is repeatedly undermined by biological complications affecting the tissues that surround them. The 2017 World Workshop on the Classification of Periodontal and Peri-Implant Diseases formalized two related entities: peri-implant mucositis, a reversible inflammatory lesion confined to the soft tissue, and peri-implantitis, in which inflammation is accompanied by progressive loss of supporting bone [1,2,3]. Epidemiological data leave little doubt about the scale of the problem. In an early and widely cited systematic review, Derks and Tomasi reported weighted mean prevalences of approximately 43% for peri-implant mucositis and 22% for peri-implantitis at the patient level [4], and later meta-analyses have arrived at broadly comparable estimates despite considerable heterogeneity in case definitions and diagnostic thresholds [5,6,7]. Because the treated population expands every year as implant therapy becomes more accessible, even a moderate prevalence translates into a substantial and growing clinical burden.

Peri-implantitis is, at its core, a biofilm-associated disease. Colonization of the implant and abutment surfaces begins within hours of exposure to the oral cavity and matures into a structured polymicrobial community whose composition shifts, in disease, toward Gram-negative anaerobic taxa [8]. The dysbiotic consortium implicated in peri-implant breakdown overlaps substantially with the periodontal “red complex” described by Socransky and colleagues—*Porphyromonas gingivalis*, *Tannerella forsythia*, and *Treponema denticola*—together with bridging orange-complex species such as *Fusobacterium nucleatum* [9]. Systematic reviews and meta-analyses of the peri-implant microbiome have confirmed the prominence of these pathogens while also stressing that the peri-implant niche is not simply a copy of the periodontal pocket: its microbial signature is heterogeneous, subject-specific, and only partially distinct from that of periodontitis [10,11,12]. This complexity has direct therapeutic consequences, because any intervention must contend with an established, matrix-protected biofilm rather than with planktonic organisms.

Current management reflects, and is constrained by, this biology. Mechanical debridement remains the cornerstone of therapy, but the very surface features that promote osseointegration also shelter bacteria from instrumentation, and vigorous cleaning can damage the implant surface and compromise its biocompatibility [2]. Adjunctive systemic or local antibiotics provide only partial and often transient benefit: they suppress commensal and pathogenic species indiscriminately, do little to restore a balanced microbiota, and add to the wider problem of antimicrobial resistance. Relapse after treatment is common [13]. These shortcomings have gradually shifted attention away from treating established disease and toward preventing colonization at the implant surface itself—and, in particular, toward surfaces that resist or actively counter bacterial adhesion without sacrificing tissue integration.

Antimicrobial peptides (AMPs) have emerged as a compelling candidate for this purpose. These short, predominantly cationic and amphipathic molecules are effectors of innate immunity in virtually all multicellular organisms, and they kill microbes principally by electrostatic association with, and disruption of, the anionic microbial membrane [14]. Because this mechanism is rapid and physical rather than directed at a single molecular target, the selective pressure for classical resistance is comparatively low, and many AMPs retain activity against biofilms and multidrug-resistant strains. Just as important for the present discussion, the biological repertoire of AMPs extends well beyond direct killing: members of the defensin and cathelicidin families modulate leukocyte chemotaxis, temper or amplify cytokine responses, and influence epithelial proliferation, migration, and angiogenesis. It is this second, immunomodulatory dimension—captured in the term “host-defense peptides”—that makes them attractive not merely as surface antiseptics but as bioactive agents capable of shaping the tissue response around an implant.

A useful and, so far, underexploited way of understanding this potential comes from skin. In the cutaneous compartment, the cathelicidin LL-37 and the human β-defensins are central not only to barrier defense but to wound repair: LL-37 is required for normal re-epithelialization and is conspicuously deficient in the epithelium of chronic, non-healing ulcers [15], while exogenous LL-37 accelerates wound closure, angiogenesis, and granulation-tissue formation in vivo [16]. The same peptides act on keratinocytes and immune cells in ways that reach far beyond their antimicrobial function [17,18]. Crucially, these are not skin-restricted molecules. The β-defensins and LL-37 are expressed in the oral mucosa, gingiva, and gingival crevicular fluid, where they take part in epithelial host defense and are dysregulated in periodontal inflammation [19,20,21]. We propose that this parallel is more than superficial: in functional terms, the transmucosal interface around an implant can be regarded as a healing epithelial barrier confronting a persistent microbial challenge—much like wounded skin. Seen through this lens, host-defense peptides offer a single mechanistic bridge between two goals usually pursued in isolation: controlling the peri-implant biofilm and promoting the soft-tissue attachment that seals the implant from the oral environment.

That soft-tissue seal is itself a decisive determinant of peri-implant health. The peri-implant mucosa forms a barrier of junctional epithelium and a supracrestal connective-tissue compartment—the peri-implant “biological width”—whose architecture and maturation have been characterized in detail in animal models [22,23,24]; a competent seal is the first obstacle to apical bacterial migration, whereas a compromised one predisposes to breakdown. Functionalizing the titanium surface with AMPs offers a way to defend this interface locally, and the available in vivo evidence is encouraging: peptide-coated implants such as those bearing GL13K achieve osseointegration comparable to that of uncoated controls, indicating that antibacterial functionalization need not come at the cost of bone integration [25]. Building on these observations, the present review synthesizes the literature on AMPs for peri-implantitis prevention through a deliberately dermatological lens. We first outline the epidemiology, microbial etiology, and limitations of current therapy; we then examine the structure and dual antibacterial–immunomodulatory activity of AMPs, the lessons that cutaneous and mucosal wound healing offer for the peri-implant context, the strategies available for engineering peptide-functionalized titanium, and the evidence that such surfaces can both reinforce the soft-tissue seal and support osseointegration. We close by appraising the translational barriers—peptide stability, cytotoxicity, manufacturing cost, regulatory demands, and the near-absence of human clinical data—that still separate a promising concept from clinical reality.

### Search Strategy and Selection Criteria

**Figure 1:**
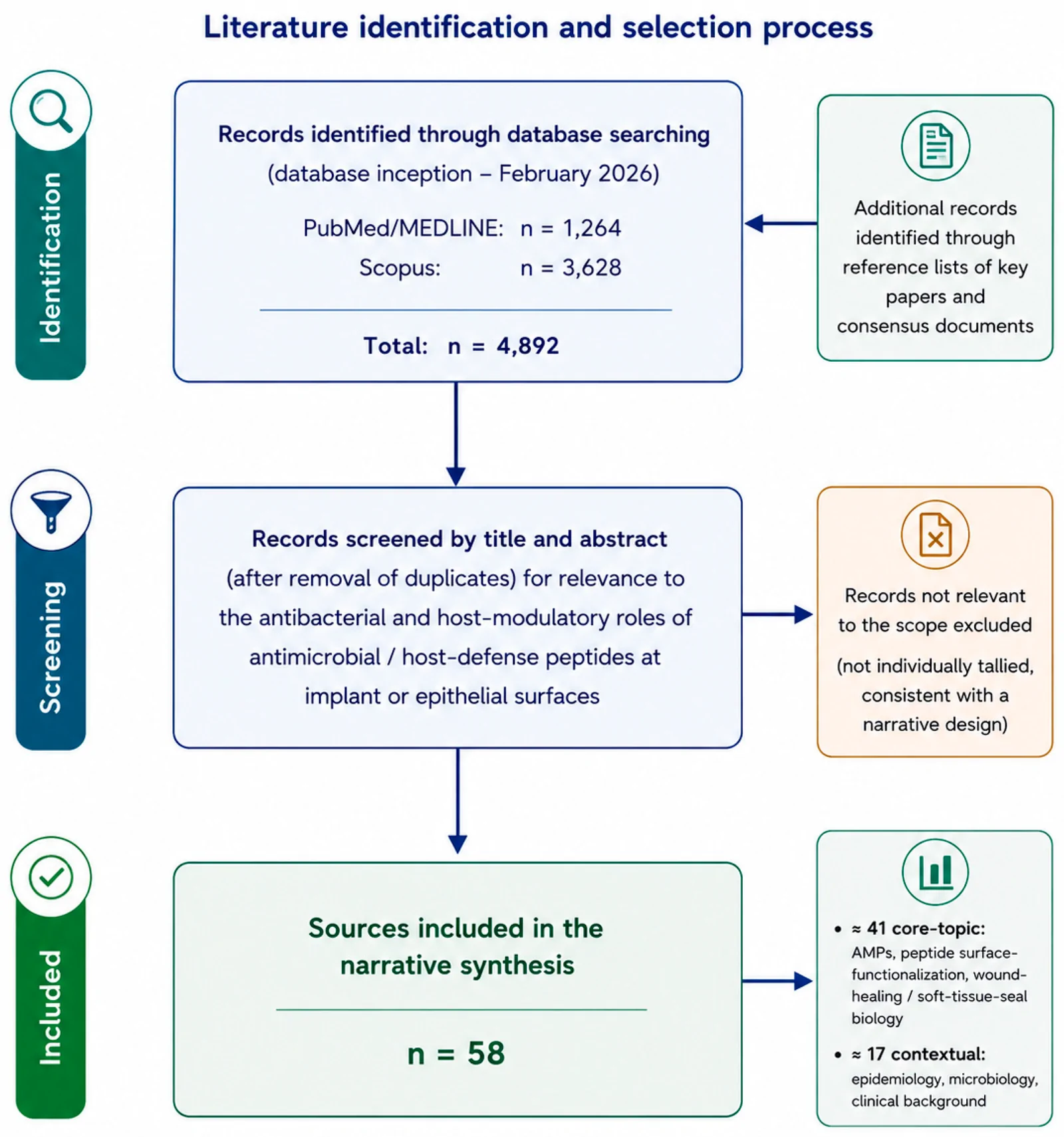
Flow diagram of the literature identification and selection process. Records were identified through PubMed/MEDLINE (*n* = 1264) and Scopus (*n* = 3628) searches (database inception–February 2026; 4892 records in total before duplicate removal), together with records identified by screening the reference lists of key papers and consensus documents. After title- and abstract-level assessment for relevance to the antibacterial and host-modulatory roles of antimicrobial and host-defense peptides at implant or epithelial surfaces, 58 sources were retained for the narrative synthesis—approximately 41 addressing peptide biology, surface-functionalization, and wound-healing and soft-tissue-seal biology, and approximately 17 providing epidemiological, microbiological, and clinical context. Consistent with the narrative design, excluded records were not individually tallied.

This article is a narrative review rather than a systematic one. The literature was identified through searches of PubMed/MEDLINE and Scopus for English-language publications up to early 2026, combining terms for antimicrobial and host-defense peptides (including LL-37, defensins, GL13K, KR-12, and HHC-36) with terms for peri-implantitis, dental implants, titanium surface functionalization, biofilm, soft-tissue seal, and osseointegration; the reference lists of key papers were screened for additional sources, covering the period from database inception to February 2026. A representative search string combined (“antimicrobial peptide” OR “host-defense peptide” OR LL-37 OR defensin OR GL13K OR KR-12 OR HHC-36) AND (peri-implantitis OR “peri-implant” OR “dental implant” OR “titanium surface” OR biofilm OR “soft-tissue seal” OR osseointegration), adapted to the syntax of each database. Eligible records were original in vitro, in vivo, and clinical studies together with authoritative reviews and consensus statements published in English; conference abstracts lacking full data, and publications unrelated to the antibacterial or host-modulatory roles of peptides at implant or epithelial surfaces, were not considered. Studies were selected for their relevance to the dual antibacterial–immunomodulatory framework developed here, with priority given to mechanistic work, surface-engineering studies, and the most recent in vivo evidence, rather than according to formal systematic-review criteria. The searches returned 1,264 records in PubMed/MEDLINE and 3628 in Scopus (database inception–February 2026; 4892 records in total before removal of duplicates), together with additional records identified by screening the reference lists of key papers and consensus documents. Consistent with this narrative and interpretive design, records were not screened against a pre-registered protocol; after title/abstract assessment for relevance to the antibacterial or host-modulatory roles of peptides at implant or epithelial surfaces, 58 sources were retained for the synthesis—approximately 41 addressing antimicrobial and host-defense peptides, peptide surface-functionalization, and wound-healing and soft-tissue-seal biology, and approximately 17 providing epidemiological, microbiological, and clinical context. The identification and selection process is summarized in Figure 1. Throughout, direct peri-implant evidence is distinguished from findings extrapolated from cutaneous wound healing, periodontal tissues, orthopaedic-implant models, or general titanium-surface studies, and the resulting framework is treated as hypothesis-generating until confirmed by standardised large-animal studies and adequately powered human clinical trials (Section 9.8). The review is therefore interpretive and conceptual in intent, and the synthesis it offers should be read as hypothesis-generating rather than as a quantitative appraisal of pooled data. The review centres on titanium and its alloys, the dominant implant and abutment substrate; because ceramic components are increasingly used in practice, however, it also considers zirconia and hybrid (titanium-base/zirconia) abutments and zirconia implants, whose distinct surface chemistry affects both peptide attachment and the mucosal seal, and these are discussed specifically in Section 7.4. To our knowledge, this is the first review to connect the cutaneous and oral host-defense biology of antimicrobial peptides specifically to the peri-implant soft-tissue seal, and to frame peri-implantitis prevention around a single class of molecules that act at once as antibacterial agents and as promoters of that seal.

## 2. Peri-Implantitis: Definitions, Epidemiology, and Clinical Burden

### 2.1. Definitions and Diagnostic Criteria

The 2017 World Workshop supplied the framework that still governs how peri-implant conditions are described. Peri-implant health is defined by the absence of clinical signs of inflammation—no erythema, no swelling, no suppuration, and no bleeding on gentle probing—around an osseointegrated implant, and it can exist on both normal and reduced bone support [1,24]. Peri-implant mucositis denotes an inflammatory lesion of the peri-implant soft tissue without accompanying loss of supporting bone; bleeding on probing is its cardinal sign, and the condition is considered reversible once the bacterial challenge is removed [1,3]. Peri-implantitis is the more consequential diagnosis: a plaque-associated pathological condition characterized by inflammation of the peri-implant mucosa together with progressive loss of supporting bone, expressed clinically as bleeding and/or suppuration on probing, increased probing depths, and radiographically detectable bone loss beyond the changes expected from initial remodelling [2].

In the common situation where baseline records are unavailable, the Workshop proposed a pragmatic case definition: the combination of bleeding or suppuration on probing, probing depths of at least 6 mm, and a bone level at least 3 mm apical to the most coronal portion of the intraosseous component is taken to indicate peri-implantitis [3]. This operational threshold is helpful at the chairside, but it also exposes a central methodological difficulty in the field. In the absence of standardized baseline radiographs and uniform thresholds, the boundary between health, mucositis, and disease is drawn differently from one study to the next—a fact that directly shapes the prevalence estimates discussed below.

### 2.2. Prevalence and Incidence

Estimates of how often peri-implant diseases occur vary considerably, yet they converge on the conclusion that both conditions are common. In their frequently cited systematic review, Derks and Tomasi calculated weighted mean prevalences of roughly 43% for peri-implant mucositis and 22% for peri-implantitis at the patient level [4]. Later meta-analyses have produced values in a comparable range while underscoring the heterogeneity of the underlying data: Lee and colleagues reported subject-level prevalences of approximately 47% for mucositis and 20% for peri-implantitis, with correspondingly lower implant-level figures [6], and a meta-analysis by Diaz and co-workers that pooled fifty-seven studies placed peri-implantitis at about 20% of patients and 13% of implants [5,7]. The most comprehensive synthesis to date—an Academy of Osseointegration/American Academy of Periodontology review of more than one hundred studies—estimated patient-level prevalences near 46% for mucositis and 21% for peri-implantitis and, importantly for clinicians counseling patients, an incidence of peri-implantitis of around 22% over follow-up periods extending toward two decades [26]. Read together, these figures suggest that roughly one in five implant patients can be expected to develop peri-implantitis over the functional lifetime of their restorations.

### 2.3. Risk Factors and Indicators

The likelihood that a given implant develops disease is modified by a recognizable set of patient- and site-level factors. A history of treated or untreated periodontitis is among the most consistently reported associations, reflecting a shared susceptibility and a shared microbial reservoir, and inadequate plaque control combined with irregular supportive maintenance compounds the risk [2,26]. Smoking and poorly controlled hyperglycemia are repeatedly implicated, and the recent AO/AAP analysis additionally identified obesity as a significant patient-level risk factor alongside periodontitis and smoking [26]. Site-specific conditions matter as well: a deficiency of keratinized peri-implant mucosa has been linked to greater plaque accumulation, discomfort during hygiene, and inflammation, and a systematic review with meta-analysis found that the absence of an adequate band of keratinized tissue was associated with a substantially higher risk of peri-implantitis [27]. Iatrogenic and prosthetic factors—residual subgingival cement, malpositioned implants, and restorations that obstruct access for cleaning—complete the picture. The common thread is that most established risk factors either increase the bacterial burden at the implant surface or impair the host’s capacity to control it, which is precisely why strategies acting at the surface itself are conceptually attractive.

## 3. Microbial Etiology and Biofilm Dynamics at the Peri-Implant Interface

### 3.1. Biofilm Formation on the Implant Surface

Bacterial colonization of an implant begins almost as soon as the surface is exposed to the oral environment. Within minutes, salivary glycoproteins adsorb to the titanium or abutment surface and form an acquired pellicle that conditions subsequent microbial attachment. The pellicle serves as a substrate for the attachment of more fastidious organisms. Early colonizers (principally streptococci and Actinomyces species) attach to it. Fusobacterium nucleatum then acts as a microbial bridge, coaggregating the initial benign community with the anaerobic species associated with disease. As the biofilm matures, its three-dimensional architecture and self-produced extracellular matrix generate localized microenvironments—oxygen gradients, nutrient niches, and protected microcolonies—that favour anaerobic, Gram-negative taxa. The very submucosal topography and surface microstructure that promote osseointegration also offer abundant sites for bacterial retention, and the coaggregation behaviour that links early and late colonizers, with *F. nucleatum* at its center, is a defining feature of this developmental sequence [9].

### 3.2. The Dysbiotic Microbiome of Peri-Implantitis

The transition from health to disease around an implant is best understood not as the arrival of a single pathogen but as a dysbiotic shift in the composition and activity of the resident community. The species repeatedly enriched at diseased sites overlap substantially with the periodontal “red complex” defined by Socransky and colleagues—*Porphyromonas gingivalis*, *Tannerella forsythia*, and *Treponema denticola*—together with orange-complex organisms such as *Fusobacterium nucleatum* and *Prevotella intermedia* [9]. Systematic reviews and meta-analyses of the peri-implant microbiome have confirmed the elevated prevalence and abundance of these anaerobes in peri-implantitis: Sahrmann and colleagues, pooling a large body of studies, reported higher detection of *Aggregatibacter actinomycetemcomitans* and *Prevotella intermedia* at diseased sites, and a more recent meta-analysis by Carvalho and co-workers reinforced the association of red- and orange-complex taxa with peri-implant breakdown [10,11]. Both syntheses, however, caution against an overly tidy picture: peri-implantitis lesions also harbor organisms less typical of classical periodontitis—opportunistic Gram-negative rods, staphylococci, and enteric species among them—which points to a broader and more variable pathogenic consortium than the periodontal model alone would predict [11,12].

### 3.3. Peri-Implant Versus Periodontal Microbiota

Although the peri-implant and periodontal microbiomes share many of the same keystone species, they are not identical, and the differences carry clinical weight. The microbial signature of peri-implantitis is consistently described as more heterogeneous and more subject-specific than that of periodontitis, and a substantial part of its diversity is only partially accounted for by the canonical periodontal pathogens [10,12,28]. Comparative analyses indicate that the peri-implant niche can sustain a distinct community structure, shaped by the abiotic titanium surface, the absence of a true periodontal ligament, the geometry of the implant–abutment connection, and the local immune environment. The practical consequence is twofold: microbiological findings from periodontitis cannot be transferred wholesale to the peri-implant setting, and interventions that target only the red-complex species may leave an appreciable share of the disease-associated community untouched [12].

### 3.4. Therapeutic Implications

Two features of peri-implant biofilms make them particularly difficult to manage and explain why conventional approaches so often fall short. First, the mature biofilm is matrix-protected: its extracellular polymeric substance limits the penetration of antiseptics and antibiotics and shelters phenotypically tolerant cells, so that concentrations achievable in vivo frequently fail to eradicate the community even when its constituent species are susceptible in planktonic culture. Second, the implant surface is at once the substrate for colonization and an obstacle to its removal, since the microstructure that favors bone anchorage resists complete mechanical decontamination and is degraded by aggressive instrumentation. Antibiotic adjuncts, for their part, act non-selectively—suppressing commensals along with pathogens without restoring a balanced community—and they carry the now-familiar risks associated with antimicrobial resistance [10,11]. These considerations point toward a different logic of intervention: one that seeks to prevent or limit colonization at the surface from the outset, ideally with an agent that resists the development of resistance and remains active within the biofilm. It is against this background that antimicrobial peptides, and surfaces functionalized with them, become a rational object of study—distinct from inorganic antiseptics and antifouling coatings in coupling antibacterial action to host-directed repair, as developed in Section 9.5—and it is to their biology that the Section 4 describes.

## 4. Antimicrobial Peptides: Structure, Mechanisms, and the Dual Antibacterial–Immunomodulatory Paradigm

### 4.1. Structural Classes and Physicochemical Properties

Antimicrobial peptides form a large and structurally diverse family, yet most share a small set of physicochemical features that underlie their activity. They are typically short—on the order of twelve to fifty amino acids—and carry a net positive charge at physiological pH, owing to an excess of lysine and arginine residues combined with a substantial proportion of hydrophobic residues. This cationic, amphipathic design allows a peptide, on contact with a membrane, to fold into a conformation in which its polar and hydrophobic faces are spatially segregated. By secondary structure, AMPs are usually grouped into a few broad classes: amphipathic α-helical peptides, of which the human cathelicidin LL-37 is the archetype; β-sheet peptides stabilized by disulfide bonds, exemplified by the defensins; and extended or loop-structured peptides enriched in particular residues. In humans, the defensins and the single cathelicidin LL-37 dominate the host-defense repertoire. The defensins are subdivided into α- and β-families according to the connectivity of their three conserved disulfide bridges; the β-defensins, including the inducible human β-defensin-3, are expressed chiefly by epithelial cells and are central to surface immunity [14,19].

### 4.2. Mechanisms of Antibacterial Action

The defining mechanism of most AMPs is direct physical disruption of the microbial membrane, and it begins with electrostatic attraction. The cationic peptide is drawn preferentially to the anionic surface of bacterial membranes—rich in negatively charged phospholipids and, in Gram-negative organisms, lipopolysaccharide—rather than to the more neutral, cholesterol-containing membranes of host cells, a difference that contributes to selective toxicity. Having accumulated at the surface above a threshold concentration, peptides insert into the lipid bilayer and permeabilize it through one of several models that are not mutually exclusive: in the barrel-stave model they form a discrete transmembrane channel; in the toroidal-pore model they bend the lipid monolayers into a continuous pore lined by both peptide and lipid head groups; and in the carpet model they accumulate parallel to the surface until the membrane disintegrates in a detergent-like manner. The outcome is loss of membrane integrity, dissipation of transmembrane gradients, and cell death. Some peptides act, in addition or instead, on intracellular targets—inhibiting nucleic-acid or protein synthesis, or interfering with cell-wall assembly—after translocating across the membrane [14].

### 4.3. Activity Against Biofilms and the Low Propensity for Resistance

Two properties make this mode of action especially pertinent to peri-implant infection. The first is activity against biofilms: because membrane disruption does not depend on active bacterial metabolism, many AMPs retain efficacy against the slow-growing, phenotypically tolerant cells that populate a mature biofilm and survive conventional antibiotics, and several peptides additionally interfere with the early stages of biofilm assembly and with intercellular signaling. The second is a comparatively low propensity to select for classical resistance. Because the target is the fundamental architecture of the microbial membrane rather than a single, mutable protein, the genetic route to resistance is less direct than for most antibiotics, and broad cross-resistance is uncommon. This is not to say that resistance is impossible—bacteria can modify membrane charge, secrete proteases, or deploy efflux systems, and resistance can be selected for under sustained exposure—but the barrier is higher, a meaningful advantage at a time of widespread antimicrobial resistance [14]. A further, more subtle concern attaches specifically to host-defense peptides: because molecules such as LL-37 and the defensins are themselves effectors of innate immunity, selecting for bacterial resistance to a therapeutic peptide could in principle erode the host’s own first-line defenses, a liability that conventional antibiotics do not carry [14].

### 4.4. The Immunomodulatory Dimension: From Antibiotics to Host-Defense Peptides

Confining the description of AMPs to their bactericidal activity would, however, miss what is arguably most relevant to implant dentistry. The defensins and LL-37 are not merely endogenous antibiotics; they are signaling molecules that shape the host response, and for this reason they are increasingly designated host-defense peptides. They recruit and activate neutrophils, monocytes, and other immune cells through chemotactic and receptor-mediated effects—LL-37 acting largely through the formyl-peptide receptor FPR2, the β-defensins through chemokine receptors such as CCR6—they modulate the production of pro- and anti-inflammatory cytokines, in some settings dampening excessive inflammation and in others amplifying a protective response; and they act directly on epithelial cells to promote proliferation and migration, on endothelial cells to support angiogenesis, and on the broader program of tissue repair [14,17,18,29]. In the skin, these activities translate into a demonstrable role in wound healing, and the same peptide families operate at oral epithelial surfaces, including the gingiva and the peri-implant mucosa [16,21]. It is this dual capacity—to kill the colonizing microbiota while simultaneously guiding the repair and sealing of the surrounding soft tissue—that defines the paradigm explored in the remainder of this review. Its therapeutic logic is seen most clearly in the biology of cutaneous and mucosal wound healing, to which the Section 5 is devoted.

## 5. Host-Defense Peptides at Epithelial Barriers: Lessons from Cutaneous and Mucosal Wound Healing

### 5.1. Cutaneous Barrier Immunity and Wound Repair

The skin offers the most thoroughly characterized model of how host-defense peptides operate at an epithelial surface, and it is from this model that the present review draws its central analogy. In healthy skin, the β-defensins and the cathelicidin LL-37 are produced by keratinocytes and contribute to a chemical barrier that limits microbial colonization, and their expression rises sharply in response to injury and infection [17,18]. What is most instructive, however, is that these peptides take part in repairing the barrier as well as defending it. LL-37 is strongly upregulated in the epithelium of healing skin wounds, and blocking its activity impairs re-epithelialization; conversely, its near-absence characterizes chronic, non-healing ulcers, in which epithelial expression of the peptide is conspicuously deficient [15]. Functional studies confirm a causal contribution: in experimental wounds, treatment with LL-37 accelerates wound closure, stimulates angiogenesis, and promotes the formation of granulation tissue, while β-defensins likewise drive keratinocyte proliferation and migration [16,17]. The broader clinical objective of promoting tissue repair while limiting infection—and of coordinating care across disciplines—is well recognized, from wound-management adjuncts such as closed-incision negative-pressure wound therapy in surgery [30] to multidisciplinary protocols in oral and maxillofacial rehabilitation [31]. The molecules that emerge from this work are valuable to the host not only because they kill microbes but because they orchestrate the cellular events that restore an intact, sealed epithelium. This dual potency is not without risk, however: LL-37 in particular is a double-edged molecule whose dysregulated or excessive expression has been implicated in inflammatory skin disorders such as psoriasis and rosacea and, in some tissues, in tumour progression, so that any therapeutic exploitation depends on keeping its local concentration within a controlled, physiological range [18].

### 5.2. Host-Defense Peptides in the Oral Mucosa and Gingiva

These are not properties peculiar to skin. The oral mucosa is, like the epidermis, a stratified epithelium under constant microbial pressure, and it deploys the same families of host-defense peptides. The human β-defensins are expressed throughout the gingival epithelium and in the salivary glands, where they form part of the innate barrier; their expression shifts with inflammation, and dysregulation accompanies periodontal disease [19,20]. LL-37 is likewise present in gingival tissue, gingival crevicular fluid, and saliva, and its importance to oral health is underscored by the clinical observation that individuals deficient in cathelicidin suffer severe, early-onset periodontal destruction; recent work has detailed both its antimicrobial action against periodontal pathogens and its immunomodulatory and tissue-regenerative roles within the periodontium [21]. The parallel between the cutaneous and oral compartments is therefore not merely structural but mechanistic: in each, the same peptides defend the surface and support the maintenance and repair of the epithelial seal.

### 5.3. The Peri-Implant Transmucosal Interface as a Healing Epithelial Barrier

**Figure 2:**
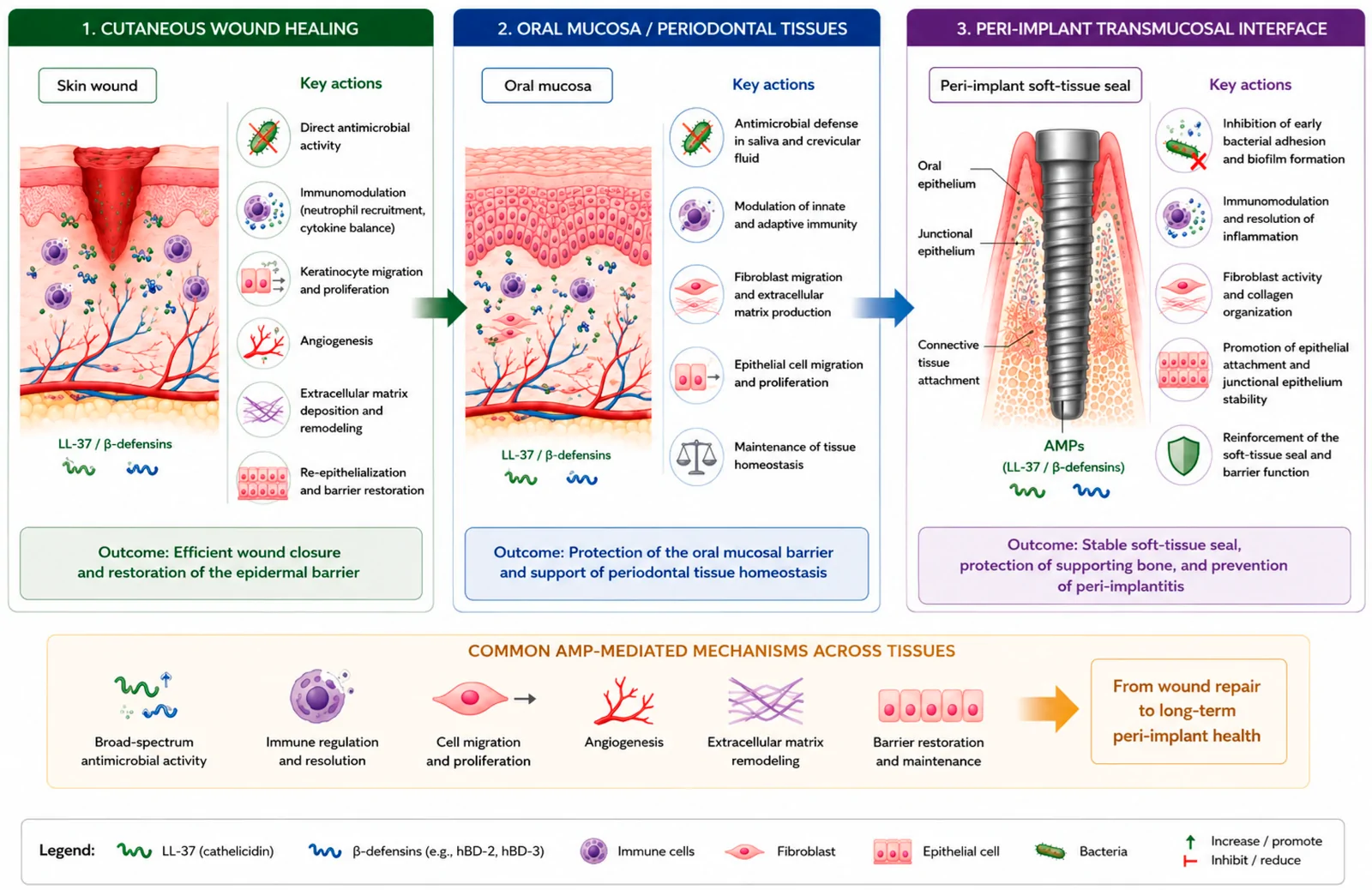
From cutaneous wound healing to the peri-implant soft-tissue seal: shared roles of host-defense peptides across the tissue continuum. Schematic comparison of LL-37 and β-defensin activity in three epithelial compartments—(1) cutaneous wound healing, (2) oral mucosa and periodontal tissues, and (3) the peri-implant transmucosal interface. In each setting, these host-defense peptides combine direct antimicrobial defense with immunomodulation, keratinocyte and fibroblast migration and proliferation, angiogenesis, extracellular matrix deposition and remodeling, and re-epithelialization and barrier restoration. The peri-implant panel highlights the soft-tissue seal—oral epithelium, junctional epithelium, and connective-tissue attachment—that these peptides help to establish and maintain. The shared repertoire of AMP-mediated mechanisms (broad-spectrum antimicrobial activity, immune regulation and resolution, cell migration and proliferation, angiogenesis, matrix remodeling, and barrier maintenance) links early wound repair to long-term peri-implant health.

This shared biology provides the conceptual bridge on which the remainder of the review rests. The basis for this convergence is mechanistic rather than merely analogical. The cathelicidin LL-37 and the β-defensins discussed above are not different molecules performing parallel roles in skin and mouth but the same host-defense peptides, produced by the same keratinocyte-derived epithelia and induced by the same signals of injury and microbial challenge across the epidermis, the gingiva, and the peri-implant mucosa. The seal itself is established through a wound-healing program—the junctional epithelium re-forms against the titanium much as the epidermis re-epithelializes a wound, while the underlying connective tissue matures through the fibroblast-driven matrix deposition and angiogenesis that these peptides promote in cutaneous repair—so that the very processes which build and sustain the seal are, at the cellular and molecular level, those of epithelial wound healing. The soft tissue surrounding a transmucosal implant must accomplish, against a persistent and dysbiosis-prone microbial challenge, precisely what wounded skin accomplishes after injury: it must establish an adherent epithelial barrier—a junctional or barrier epithelium supported by a connective-tissue zone—that seals the underlying bone and implant from the oral environment. In functional terms, the peri-implant mucosa behaves like a healing epithelial wound that never fully closes around a foreign, non-shedding surface, and throughout its life it is exposed to the same pathogens as the gingiva and the skin. Seen through this lens, the same host-defense peptides that safeguard the skin and oral mucosa emerge as natural candidates to protect the peri-implant transmucosal interface (Figure 2). It must be stressed, however, that this convergence is at present a conceptual framework rather than a demonstrated equivalence: direct evidence for host-defense peptide activity at the peri-implant interface itself remains limited, and much of the rationale developed here is extrapolated from the better-studied cutaneous and oral compartments. The analogy also has clear limits. The skin is a keratinized, continuously desquamating epithelium endowed with appendages and its own distinct microbiota, whereas the peri-implant mucosa abuts a hard, non-shedding titanium surface, lacks the rete-ridge interlocking of the epidermis and, unlike a natural tooth, is deprived of a periodontal ligament and of connective-tissue fibres inserted perpendicular to the surface [32]. Lessons drawn from cutaneous repair must therefore be transferred with caution rather than assumed to apply unchanged. A surface that presents host-defense peptides, or engineered analogues of them, could in principle reduce bacterial colonization while simultaneously encouraging the epithelial and connective-tissue responses that constitute the seal. Several naturally occurring and synthetic peptides have been explored with exactly this dual intent, among them GL13K, a cationic peptide derived from a human salivary protein and studied extensively as a titanium coating [25], and short cathelicidin-derived fragments such as the LL-37-based KR-12 peptides, valued for combining antibacterial potency with favorable, anti-inflammatory effects on host cells [33,34]. Translating the concept into a clinical device, however, requires that the peptide be stably attached to the titanium surface, retain its activity in the oral environment, and act without harming the very cells it is meant to support—the engineering challenges examined in the Section 6.

## 6. Engineering AMP-Functionalized Titanium Implant and Abutment Surfaces

### 6.1. Immobilization Strategies: Covalent Attachment and Layer-by-Layer Assembly

Translating the biology of host-defense peptides into a working implant surface depends, before anything else, on how the peptide is fixed to the titanium. The immobilization strategies described below apply, in principle, equally to the endosseous body and to the transmucosal (abutment or collar) surface on which the soft-tissue seal forms; where the distinction matters for peri-implant disease it is developed in Section 7.4. Two broad philosophies have emerged. In contact-killing designs, the peptide is immobilized on the surface and kills bacteria that come into direct contact with it, ideally providing a durable, non-depleting antibacterial effect; in release-based designs, the peptide is held in or on the surface and diffuses outward to act in the surrounding tissue. Covalent attachment is the principal route to a stable contact-killing coating [35,36]. Peptides are commonly anchored to titanium through silane coupling agents that bridge the metal-oxide surface and a reactive group on the peptide, and refinements of this chemistry have improved both the efficiency and the selectivity of grafting: a chemoselective approach that couples a cysteine-terminated GL13K peptide to the surface produces a coating that remains stable, retaining its surface signal and antibacterial function even after mechanical and chemical challenge [37]. Layer-by-layer assembly offers an alternative, in which oppositely charged polyelectrolytes and peptide are deposited in successive layers; using this technique, a broad-spectrum peptide coupled to a collagen scaffold has been assembled on titanium, reducing the growth of bacteria responsible for peri-implantitis and inhibiting biofilm formation on the surface [38].

### 6.2. Controlled Release and Carrier Systems

Where a sustained, diffusible dose is desired—for instance, to protect the surface during the vulnerable early healing period—carrier systems provide control over how much peptide is released and for how long. Nanoparticle carriers are particularly versatile. In one well-developed example, the antimicrobial peptide HHC-36 was loaded into diselenide-bridged mesoporous silica nanoparticles immobilized on titanium, producing a surface that released the peptide in a controlled fashion over roughly thirty days, maintained mechanical stability, and achieved high antibacterial activity against several clinically relevant species while also favorably modulating inflammation and osseointegration in vivo [39]. Stimulus-responsive carriers extend this logic further, coupling release to the local conditions of infection: hydrogels and nanostructures designed to respond to the acidic microenvironment of an infected site can concentrate peptide delivery where and when it is needed, aligning the antibacterial action with the biological cues of the peri-implant wound [40].

### 6.3. Coating Stability and the Osseointegration Constraint

Whatever the immobilization strategy, two requirements constrain any candidate coating. The first is durability, both mechanical and biochemical. A coating must survive the considerable shear forces generated as an implant is threaded into bone, and it must resist degradation by the proteases and dynamic chemistry of the oral environment; a surface that loses its peptide load or activity within days cannot prevent a disease that develops over months and years. Covalent, well-anchored systems have been shown to retain their integrity under such challenges [37]. The second requirement is biocompatibility, and it is the more delicate of the two, because the peptide must be cytotoxic to bacteria without harming the host cells responsible for integration. The same coating is expected to permit, and ideally to promote, the attachment and function of osteoblasts at the bone interface and of epithelial cells and fibroblasts at the transmucosal collar. Reassuringly, peptide-functionalized titanium can meet this dual standard: GL13K-coated implants achieve osseointegration comparable to that of uncoated controls in vivo, indicating that the antibacterial benefit need not be purchased at the cost of bone integration [25].

### 6.4. Representative Peptides and Rational Design

A relatively small number of peptides recur across this literature, each illustrating a different design consideration. GL13K, derived from a human salivary protein, has become the most extensively studied dental-implant peptide and shows that a covalently bound coating can combine durability, antibacterial activity, and compatibility with bone [25,37]. HHC-36 exemplifies the carrier-based, controlled-release strategy [39], while the cathelicidin-derived KR-12 peptides illustrate the appeal of short, LL-37-based fragments that pair antibacterial potency with anti-inflammatory effects on host cells [33]. Increasingly, the choice of peptide is being placed on a rational footing: rather than screening natural sequences empirically, computational and machine-learning methods are now used to design peptides with optimized activity and selectivity, including sequences tailored to target the progression of peri-implant disease while sparing commensal species [41]. Such approaches point toward a future in which the coating is engineered not merely for stability and potency but for a defined ecological effect on the peri-implant community—a theme taken up again in the concluding section.

These representative systems, and the strategies they embody, are summarized in Table 1.

## 7. Reinforcing the Peri-Implant Soft-Tissue Seal with Bioactive Peptides

### 7.1. Biology of the Peri-Implant Soft-Tissue Seal

The barrier that forms between the oral cavity and the underlying bone is the implant’s first and most exposed line of defense, and its structure differs in important ways from the attachment around a natural tooth. The peri-implant mucosa comprises a junctional or barrier epithelium, continuous with the oral epithelium, supported by a zone of supracrestal connective tissue; together these constitute the peri-implant biological width [22,24]. Experimental work has traced how this barrier develops and matures: the junctional epithelium reaches a length of roughly two millimetres and the connective-tissue compartment organizes over several weeks after the mucosa is connected to the implant [23]. Yet the peri-implant seal is, in several respects, weaker than that around a tooth. Lacking a periodontal ligament and the perpendicular insertion of connective-tissue fibres into cementum, the peri-implant connective tissue runs largely parallel to the surface, contains more collagen and fewer fibroblasts, and is less richly vascularized—features that render the seal more vulnerable to mechanical disruption and bacterial ingress. A deficient soft-tissue compartment is recognized as a contributor to peri-implant pathology, and strategies that strengthen the attachment of soft tissue to the transmucosal surface are therefore of direct clinical interest [46].

### 7.2. The “Race to the Surface” at the Transmucosal Region

**Figure 3:**
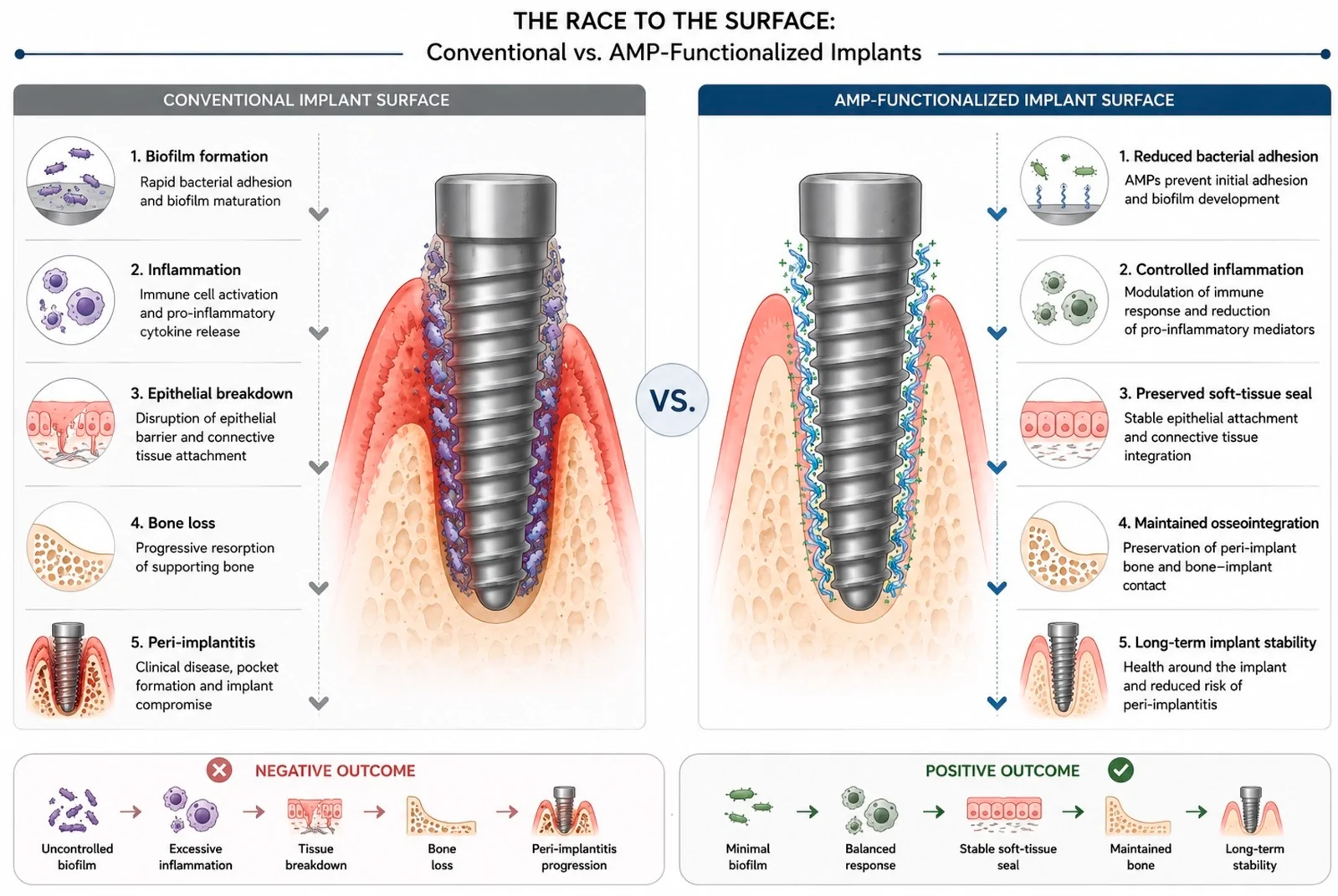
The race to the surface: conventional versus AMP-functionalized implant surfaces. Conceptual comparison between conventional implant surfaces and antimicrobial peptide (AMP)-functionalized implant surfaces according to the “race to the surface” paradigm. Conventional implants are characterized by early bacterial adhesion, biofilm maturation, excessive inflammation, disruption of the epithelial and connective tissue attachment, progressive peri-implant bone loss, and eventual peri-implantitis. In contrast, AMP-functionalized surfaces reduce bacterial colonization, modulate local inflammatory responses, preserve the peri-implant soft-tissue seal, maintain osseointegration, and support long-term implant stability. The figure illustrates how host-cell integration and microbial competition at the transmucosal interface determine peri-implant health outcomes.

A useful way to frame the problem is the concept of a “race to the surface,” in which host cells and bacteria compete to occupy a newly placed implant. If host cells—gingival fibroblasts and epithelial cells—colonize and integrate with the transmucosal surface first, a protective seal is established; if bacteria reach and colonize the surface first, a biofilm forms and integration is compromised [47]. This framing has a clarifying consequence for surface design. Rather than treating antibacterial protection and soft-tissue integration as separate objectives, it suggests that the ideal transmucosal surface should tip the race in both directions at once—actively favouring the adhesion and activity of host cells while simultaneously discouraging or killing the colonizing microbiota. It is precisely this combination that bioactive peptides are positioned to deliver, as summarized in Figure 3.

### 7.3. Peptide Strategies to Reinforce the Seal

Two complementary peptide approaches have been pursued. The first uses peptides engineered specifically to anchor host cells to titanium. By combining titanium-binding sequences selected through phage display with cell-adhesion motifs derived from laminin-5 and E-cadherin, bifunctional metal–cell-specific peptides can act as molecular linkers between the implant surface and the epithelium; in a rat model, a surface functionalized with such a peptide not only supported stable cell adhesion on the transgingival portion of the implant but also arrested the unwanted apical migration of epithelial cells, producing a shorter junctional epithelium than the bare alloy [45]. This approach addresses the sealing problem directly, though it does not, by itself, confer antibacterial activity.

**Figure 4:**
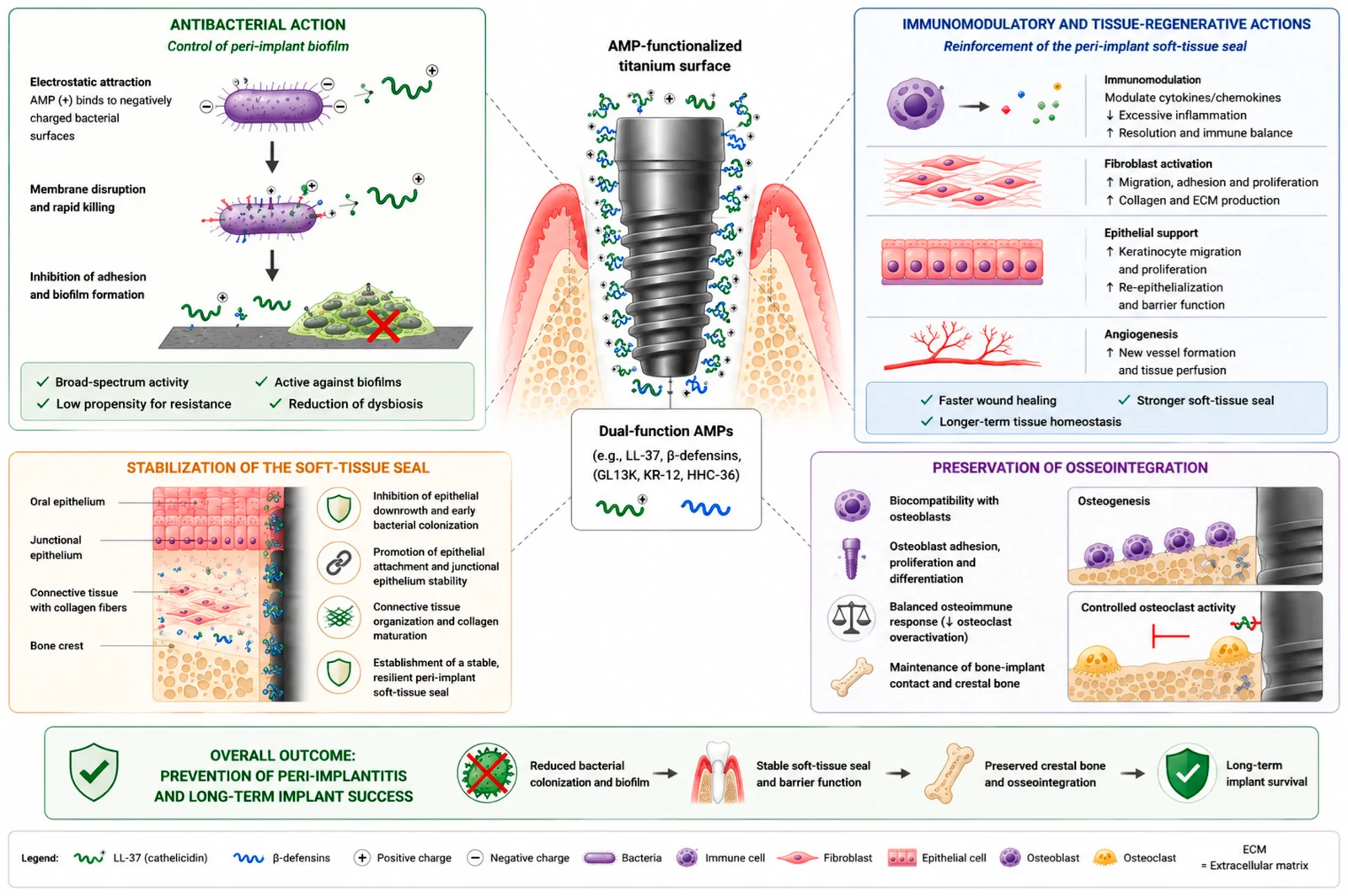
AMP-functionalized implant surfaces: integrated antibacterial, immunomodulatory, and tissue-regenerative mechanisms for peri-implantitis prevention. Schematic overview of the biological mechanisms through which antimicrobial peptide (AMP)-functionalized titanium surfaces may prevent peri-implant disease while preserving tissue integration. Following immobilization on the implant surface, AMPs exert direct antibacterial effects by disrupting bacterial membranes, inhibiting microbial adhesion, and suppressing biofilm formation. Host-defense peptides such as LL-37, β-defensins, GL13K, KR-12 and HHC-36 also modulate local immune responses, enhance fibroblast migration and extracellular matrix production, stimulate epithelial proliferation and barrier restoration, and promote angiogenesis. These effects contribute to formation and stabilization of the peri-implant soft-tissue seal, limiting bacterial penetration towards deeper tissues. At the bone interface, AMP-functionalized surfaces preserve osteoblast viability and differentiation, modulate osteoimmune responses, and preserve osseointegration without impairing implant integration. The combination of antibacterial, pro-healing and osteogenic functions may result in a reduction in peri-implant biofilm formation, an increase in soft-tissue attachment, preservation of crestal bone and an improvement of long-term implant survival.

The second approach is the more complete realization of the thesis advanced in this review, because it uses a single host-defense peptide to accomplish both tasks. In a recent study, the cathelicidin LL-37—the same peptide central to cutaneous wound healing—was immobilized on a nanostructured titanium surface through a polydopamine intermediate layer and released in a sustained manner over more than a week. The resulting surface displayed a genuine dual functionality: it significantly promoted the migration, adhesion, proliferation, and extracellular-matrix synthesis of human gingival fibroblasts, while exerting potent antibacterial activity against Porphyromonas gingivalis and Streptococcus mutans. Most tellingly, in a rat model of immediate post-extraction implantation, the LL-37-functionalized surface produced, after four weeks, a peri-implant epithelial structure resembling the junctional epithelium of a natural tooth and a soft-tissue seal that resisted the penetration of a tracer molecule [44]. This is, in effect, a proof of concept for the dermatological logic developed earlier: a peptide whose role in healing wounded skin is well established, transferred to the surface of a dental implant, simultaneously suppresses the peri-implant pathogens and builds the soft-tissue seal that protects against them. The convergence of antibacterial and pro-sealing function in a single molecule is the strongest evidence to date that the two goals of implant-surface design need not be pursued apart, although it rests, for now, on a single proof-of-concept study that awaits independent replication (Figure 4).

### 7.4. The Transmucosal Target: Implant Body, Collar, or Abutment—And the Role of Ceramic Surfaces

A distinction held in the background of the preceding discussion must now be made explicit, because it determines where on the implant–abutment complex a host-defense peptide would most usefully act. Peri-implant mucositis and peri-implantitis are not primarily diseases of the buried, osseointegrated implant body but of the transmucosal region: the inflammatory lesion begins at the mucosal margin and, in peri-implantitis, advances apically along the transmucosal surface toward the crestal bone, typically months or years after the prosthesis has been loaded. In a two-piece system, the surface that traverses the mucosa and against which the junctional epithelium and supracrestal connective tissue establish their seal is not the endosseous fixture but the abutment (or the prosthetic transmucosal component); in a one-piece or tissue-level implant it is the polished transmucosal collar of the fixture itself. It follows that, for the soft-tissue-sealing function placed at the centre of this review, the surface most in need of host-defense-peptide functionalization is the transmucosal one—the abutment or collar in contact with the peri-implant mucosa—rather than the intraosseous portion, whose functionalization is relevant chiefly to protecting the surface during early healing and to osseointegration (Section 8).

These two roles are complementary rather than competing. An antibacterial, pro-sealing coating on the transmucosal component acts at the site where disease actually initiates and where the epithelial and connective-tissue attachment must be maintained, while an osteoconductive, antibacterial treatment of the endosseous body protects primary integration. Reassuringly, most of the immobilization chemistries reviewed here—silanization, polydopamine intermediate layers, layer-by-layer assembly, and nanoparticle carriers—are in principle as applicable to a machined transmucosal surface as to an endosseous one; and the LL-37 proof-of-concept discussed above is, in fact, already a transmucosal demonstration, since the junctional-epithelium-like seal formed against the peptide-bearing surface exposed to the soft tissue rather than against bone [44]. Reframing the target in this way therefore sharpens, rather than weakens, the argument developed throughout this article.

This transmucosal emphasis intersects with a well-recognized prosthetic principle. Repeated disconnection and reconnection of the abutment during the restorative phase mechanically disrupts the maturing mucosal seal and provokes apical migration of the connective-tissue attachment with attendant marginal bone loss; a meta-analysis of abutment disconnection/reconnection found significantly greater peri-implant bone loss when the abutment was repeatedly manipulated [48]. The “one abutment–one time” protocol—placing the definitive abutment at the time of surgery and never removing it—was advanced precisely to preserve this seal, and systematic-review evidence supports a marginal-bone-level benefit relative to repeated abutment changes [49]. A transmucosal surface that actively fostered epithelial and connective-tissue attachment while suppressing colonization would be most valuable in exactly this setting, where the seal, once established, is intended to remain undisturbed for the functional lifetime of the restoration. More recent evidence is consistent: a 12-month randomized trial found that placing the definitive abutment once and leaving it undisturbed reduced radiographic marginal bone loss relative to repeated disconnection and reconnection [50], and a systematic review and meta-analysis likewise associated abutment configurations that limit repeated manipulation with less marginal bone loss [51].

Two further points follow from taking the transmucosal component as the target. First, its material is not confined to commercially pure titanium or its alloys. Zirconia (yttria-stabilized tetragonal zirconia polycrystal) abutments, and hybrid abutments that combine a titanium bonding base with a zirconia superstructure, are now widely used; comparative clinical evidence indicates that they support a peri-implant soft-tissue attachment broadly comparable to titanium, with advantages in mucosal colour and, in several studies, a tendency toward less inflammation and lower bacterial adhesion [52,53]. Zirconia dental implants themselves, though still a minority of the market, continue to gain ground as a metal-free alternative [53]. Second, the surface chemistry of zirconia differs materially from that of titanium: it lacks both the abundant surface hydroxyl groups and the native titanium-dioxide layer that silane- and polydopamine-based grafting exploit, so the immobilization strategies optimized for titanium cannot be transferred unchanged, and peptide attachment to zirconia requires its own tailored chemistry. The host-defense-peptide rationale advanced here is, however, material-agnostic at the biological level: whatever the substrate, the objective is a transmucosal surface that simultaneously discourages the dysbiotic biofilm and encourages the epithelial and connective-tissue seal. Extending peptide functionalization from titanium to zirconia and hybrid transmucosal components—and comparing the quality of the mucosal seal achieved on each—is therefore a natural, clinically pertinent direction for the future work outlined in Section 9. Recent systematic reviews and meta-analyses reinforce this position: abutment material exerts only a limited influence on peri-implant soft-tissue and marginal-bone outcomes, with zirconia and titanium performing comparably while zirconia offers advantages in soft-tissue colour [54,55]; in vitro, zirconia surfaces tend to accumulate less biofilm than titanium under comparable conditions [56]; and contemporary zirconia implants achieve survival and marginal-bone outcomes approaching those of titanium [57,58].

## 8. In Vivo Evidence and Osseointegration Outcomes

### 8.1. Osseointegration of AMP-Functionalized Implants in Animal Models

The central concern raised by any antibacterial surface modification is whether it interferes with the bone integration on which implant success depends, and the in vivo evidence on this point is reassuring. In a rabbit femoral-condyle model, implants coated with GL13K achieved bone-to-implant contact and peri-implant bone volume comparable to those of uncoated controls, as assessed by micro-computed tomography and histomorphometry, demonstrating that a covalently bound antibacterial peptide need not impair early osseointegration [25]. Carrier-based systems have produced similar or more favourable results. The HHC-36-releasing mesoporous-silica surface described earlier was evaluated in a rabbit bone-defect model, where it not only sustained antibacterial activity but supported bone regeneration and modulated the local inflammatory response, indicating that controlled peptide release can be combined with osteoconductive performance [39]. Across these studies the recurring finding is that peptide functionalization, whether by stable immobilization or by controlled release, preserves—and in some cases enhances—bone integration rather than compromising it.

### 8.2. The Osteoimmunological Dimension

Why peptide coatings might actively favour bone integration becomes clearer when osseointegration is viewed as an immune-mediated process. It is now widely recognized that the early response of innate immune cells—particularly the polarization of macrophages between pro-inflammatory and reparative phenotypes—governs the subsequent balance of osteogenesis, osteoclastogenesis, and angiogenesis at the implant surface. Host-defense peptides, with their combined antibacterial and immunomodulatory character, are well placed to influence this microenvironment. GL13K immobilized on titanium through silanization is biocompatible with bone-marrow stromal cells, endothelial cells, and macrophages, and in co-culture it fosters a pro-regenerative immune environment that promotes late-stage osteogenic differentiation, reflected in upregulated type I collagen expression and increased extracellular-matrix mineralization [42]. The same peptide also acts on the resorptive side of bone turnover: GL13K-modified titanium partially inhibits osteoclast differentiation by limiting the formation of the actin ring required for resorption and by downregulating osteoclast-associated genes, an effect traced to epigenetic methylation of the *NFATc1* promoter following RANKL stimulation [43]. Together these findings suggest that GL13K does more than spare bone—it shifts the osteoimmune balance toward formation and away from excessive resorption, a property directly continuous with the immunomodulatory paradigm introduced earlier.

### 8.3. Synthesis of the Evidence Base

Taken as a whole, the available preclinical evidence indicates that antimicrobial peptide-functionalized titanium surfaces provide simultaneous antibacterial activity and preservation of osteogenic potential, while several in vivo studies have demonstrated maintenance or enhancement of osseointegration in animal models [25,39,42,43]. Importantly, such coatings are generally cytocompatible not only with osteoblasts but also with the gingival fibroblasts on which the transmucosal seal depends [59], indicating that the antibacterial benefit need not come at the expense of either bone or soft-tissue integration. This encouraging picture must, however, be read with its limitations in mind. The great majority of the available evidence comes from in vitro experiments and small-animal models rather than from clinical studies; the peptides, loading methods, substrate textures, and bacterial challenges differ widely between investigations, which limits direct comparison; and a minority of studies report a modest increase in cytotoxicity toward osteoblast-like cells, a reminder that the therapeutic window between antibacterial efficacy and host-cell safety must be established for each system. These caveats mark the distance between a promising body of preclinical work and a clinically usable device, and they are the subject of the Section 9.

Table 2 contrasts the four most extensively studied peptides across the dimensions that are central to this review, making it explicit where each combines antibacterial and host-directed activity and where evidence is still lacking.

## 9. Translational Barriers and Future Perspectives

### 9.1. Proteolytic Stability and Strategies to Overcome It

The properties that make antimicrobial peptides attractive are accompanied by liabilities that have so far limited their clinical deployment, and chemical instability is foremost among them. Natural AMPs are composed of L-amino acids and are therefore susceptible to proteolytic degradation, with correspondingly short half-lives—a particular concern in the oral cavity, an environment rich in host and bacterial proteases [60]. Immobilizing a peptide on the implant surface, especially by covalent attachment, partially mitigates this problem by restricting access of proteases and anchoring the peptide in place, which is one of the arguments for the surface-engineering strategies discussed in Section 6. At the molecular level, a range of modifications can further enhance stability: substitution with D-amino acids or non-natural residues, backbone cyclization, PEGylation, and lipidation all reduce susceptibility to proteolysis [61]. Each of these gains, however, tends to come at a price—reduced antimicrobial potency, altered selectivity, or higher manufacturing complexity—so that stabilization must be balanced against activity for any given application [60].

### 9.2. Cytotoxicity and the Therapeutic Window

A second constraint is host-cell toxicity. Although the selectivity of cationic peptides for anionic microbial membranes limits their effect on mammalian cells, this selectivity is not absolute, and at higher concentrations many AMPs are cytotoxic or haemolytic [60]. For an implant surface, the relevant requirement is a therapeutic window wide enough that the peptide remains bactericidal at concentrations that osteoblasts, fibroblasts, and epithelial cells tolerate. The observation that some peptide-functionalized titanium surfaces can produce a modest increase in cytotoxicity toward osteoblast-like cells is a concrete reminder that this window cannot be assumed and must be characterized for each peptide, dose, and presentation. Engineering approaches that lower toxicity—PEGylation, for instance—are available, but they frequently reduce antimicrobial activity as well, which returns the problem to the same balancing exercise described above.

### 9.3. Manufacturing Cost and the Regulatory Pathway

Beyond biology lie practical and regulatory obstacles. The synthesis of peptides, typically by solid-phase methods, is costly relative to small-molecule antibiotics, and the very modifications that improve stability—D-amino acids, cyclization—increase the cost further and complicate scale-up [60]. A peptide-functionalized implant, moreover, is not a simple medical device but a combination of a device and a biologically active molecule, a status that brings demanding requirements for the demonstration of safety, efficacy, and manufacturing consistency. It is partly for these reasons that, despite decades of research, relatively few antimicrobial peptides have reached clinical approval, and none, to date, in the specific form of a functionalized dental implant; the magainin-derived peptide pexiganan, whose topical formulation failed to demonstrate superiority over its vehicle in two Phase III trials for mildly infected diabetic-foot ulcers, is a sobering reminder that strong in vitro activity does not guarantee clinical benefit [60]. Beyond approval, several practical questions bear on whether such a surface could enter routine production. The terminal sterilization applied to implants—gamma irradiation, electron-beam, or ethylene oxide—can degrade or denature surface-bound peptides, so a compatible sterilization route, or validated aseptic processing, must be established for each construct. Peptide-functionalized surfaces also raise questions of shelf-life and storage stability, of reproducible large-scale grafting under good-manufacturing-practice conditions, and of how an additional coating step could be integrated, at acceptable cost, into established implant-production workflows. None of these obstacles is insurmountable, but together they add a development burden that purely mechanical or inorganic surface treatments do not carry [60].

### 9.4. The Clinical Evidence Gap

The most consequential gap, however, is evidential. As the Section 9.3 pointed out, the case for AMP-functionalized titanium rests almost entirely on in vitro experiments and small-animal models, with clinical studies essentially absent from the field. There is, at present, no body of human data—let alone randomized controlled trials—establishing that an AMP-coated implant reduces the incidence of peri-implantitis or improves long-term survival in patients. Closing this gap will require not only first-in-human studies but also a degree of standardization that the preclinical literature currently lacks, so that peptides, coating methods, and outcome measures can be compared across investigations rather than each study standing alone.

### 9.5. Future Perspectives: Rational Design and Multifunctionality

It is worth situating peptides within the broader landscape of antibacterial implant surfaces. Inorganic approaches—silver, copper, and other metal-ion or nanoparticle coatings—are broadly bactericidal but act non-specifically and can be cytotoxic to host cells at the concentrations required for efficacy, without contributing to tissue repair. Antifouling chemistries such as poly(ethylene glycol) and zwitterionic polymers lower bacterial adhesion, yet the same protein- and cell-resistant character that makes them antifouling tends to leave them bioinert, doing little to encourage the osteoblast and epithelial integration on which the implant depends. Grafted conventional antibiotics, for their part, offer only a finite reservoir, a narrow spectrum, and the very resistance liability that motivated the search for alternatives. Against this background, a recent systematic review concluded that peptide coatings are the most extensively studied and, to date, among the most effective and biocompatible options for controlling colonization of dental-implant surfaces over prolonged periods [62]. What sets them apart is not antibacterial potency alone but its combination with host-directed, pro-healing activity—the property this review has placed at its centre. Several converging directions nonetheless make this a promising rather than a discouraging picture. The first is rational design. Where early work relied on screening natural sequences, computational and machine-learning methods now permit peptides to be designed for defined properties—optimized potency, improved selectivity, and even a targeted ecological effect, such as suppressing keystone pathogens while sparing commensal species in the peri-implant community [41]. This ecological dimension deserves emphasis. Peri-implant health is not sterility of the surface but a balanced microbial community; a broad-spectrum coating that indiscriminately suppressed colonization could, in principle, deplete the protective commensal species whose presence helps resist overgrowth by pathogens, much as broad-spectrum antibiotics disturb the gut flora. An ideal coating would therefore be ecologically selective—curbing the dysbiotic, predominantly Gram-negative anaerobic consortium associated with disease while sparing the commensal flora compatible with health [9,41]. How AMP-functionalized surfaces reshape the peri-implant microbiome as a whole, rather than the few model pathogens usually tested, is at present almost unstudied and is an important question for future work. The second is multifunctionality. The most compelling surfaces reviewed here do more than one thing at once: they combine antibacterial action with controlled release [39], with reinforcement of epithelial attachment [45], or, in the case of LL-37-functionalized titanium, with active promotion of the soft-tissue seal [44]. Designing constructs that deliberately integrate antibacterial, pro-sealing, and osteogenic functions—whether in a single peptide or in a rationally combined system—is a natural next step. The third direction is the one that motivates this review: the deliberate repurposing of host-defense peptides whose roles in epithelial repair are already understood from dermatology and mucosal biology, such as LL-37 and its derivatives [33,44]. Read together, these strands suggest that the future of antibacterial implant surfaces lies not in ever-stronger antiseptics but in biologically intelligent coatings that defend the surface and help build the tissue that seals it—the unifying idea developed throughout this article and summarized in the conclusions that follow.

### 9.6. Emerging Technologies: Toward Intelligent, Personalized Peptide Coatings

Beyond the rational-design and multifunctionality principles outlined above, several rapidly maturing technologies are poised to shape the next generation of AMP-functionalized implants. The most immediate is the application of artificial intelligence and machine learning to peptide discovery. In place of the slow, empirical screening of natural sequences, deep-learning models trained on large peptide libraries can now predict antibacterial potency, host-cell selectivity, and proteolytic stability directly from sequence, while generative algorithms propose entirely novel sequences optimized for several objectives at once [41]. Applied to the peri-implant setting, such in silico screening could compress a historically protracted search into a guided pipeline that advances only the most promising candidates to synthesis and biological validation, and could explicitly co-optimize the antibacterial and pro-healing properties placed at the centre of this review.

A second direction is the shift from static coatings toward smart, stimulus-responsive surfaces. Most of the coatings reviewed here present or release their peptide at a constant rate, yet the bacterial challenge to an implant is episodic rather than continuous. Surfaces engineered to release peptide on demand—triggered by the local fall in pH, the proteolytic activity, or the quorum-sensing molecules that accompany bacterial colonization—would concentrate antimicrobial action precisely when and where it is needed, conserving the finite peptide reservoir and limiting unnecessary exposure of host cells. Coupling peptide presentation to the biochemical signature of incipient infection is a particularly attractive way to extend the functional lifetime of a coating across the months and years over which peri-implantitis develops [39].

Closely related is the integration of peptides with nanomaterials. Conjugating AMPs to nanoparticles, or co-delivering them from nanostructured carriers, can shield the peptide from proteolysis, raise its effective local concentration, and add complementary functions—for instance pairing the host-directed, pro-healing activity of a host-defense peptide with the broad bactericidal reach of a metal-ion or nanoparticle component within a single construct [39,63]. Such peptide–nanoparticle hybrids offer a route to uniting the selectivity of peptides with the robustness of inorganic antimicrobials while mitigating the cytotoxicity that constrains the latter when used alone.

Finally, the ecological view developed earlier points toward personalized modulation of the peri-implant microbiome. As chairside microbial-profiling and sequencing tools become more accessible, surface chemistry—or an accompanying, locally delivered agent—could in principle be tailored to an individual patient’s microbial risk profile, curbing the dysbiotic, predominantly Gram-negative anaerobic consortium associated with disease while preserving the commensal community compatible with health [9,41]. None of these directions removes the need for the standardized, clinically anchored evaluation set out below; taken together, however, they mark a conceptual shift from peptides as passive antiseptic films toward intelligent, patient-adapted biointerfaces that defend the surface and actively support the tissue that seals it.

### 9.7. Knowledge Gaps and Research Priorities

Bringing this body of work together, it is useful to state plainly where the principal gaps lie, since they define the agenda for the next phase of research. The overarching one—the near-total absence of clinical evidence set out in Section 9.4—frames all the others: until controlled studies adopt peri-implant disease, rather than a surrogate such as in vitro bacterial reduction, as their endpoint, the approach will remain a biologically plausible hypothesis rather than an evidence-based therapy. Beyond it, three more specific and tractable gaps stand out.

A second gap is the lack of direct, head-to-head comparison between candidate peptides. The literature reviewed here is largely a collection of single-peptide, single-laboratory studies that differ in substrate texture, immobilization chemistry, peptide density, and bacterial challenge, which makes it difficult to judge whether GL13K, an LL-37 fragment such as KR-12, or a rationally designed peptide such as HHC-36 is best suited to the peri-implant environment. Table 3 collates the quantitative parameters reported for these leading peptides and makes plain how heterogeneous—in assay format, target organism, and reported units—the available data remain, which is itself an obstacle to ranking them. Standardized comparative studies—ideally testing several peptides on a common surface, against a common and clinically representative polymicrobial biofilm and a common panel of host cells—are needed before any candidate can be prioritized for clinical development; such studies should report comparable quantitative endpoints—minimum inhibitory concentrations, biofilm-inhibition rates, peptide-release kinetics, host-cell cytotoxicity thresholds, soft-tissue responses, and osseointegration outcomes—so that the platforms can be ranked rather than merely catalogued.

A third gap concerns durability. Peri-implantitis develops over months and years, yet almost all coating studies report activity over days to a few weeks; there is essentially no information on whether a peptide coating remains both bactericidal and pro-regenerative after one, three, or five years of exposure to the mechanical and enzymatic stresses of the oral cavity, nor on how the surface behaves once the peptide is eventually depleted. Long-term durability data, together with a clear understanding of the fate of the coating over the functional lifetime of an implant, are prerequisites for any claim of lasting protection.

**Figure 5:**
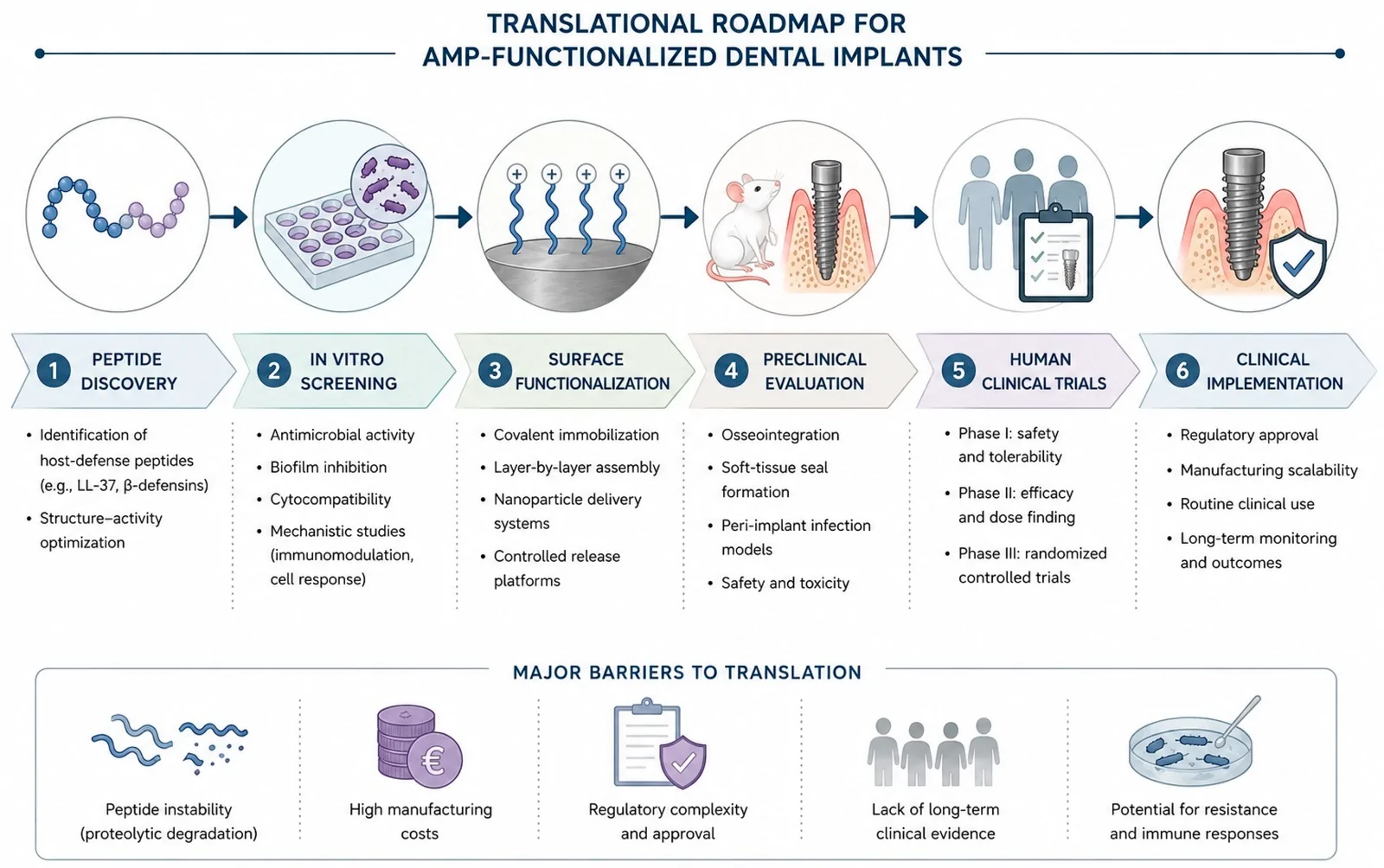
Translational roadmap for antimicrobial peptide-functionalized dental implants. Proposed translational pathway for antimicrobial peptide (AMP)-functionalized dental implants from peptide discovery to routine clinical implementation. The roadmap illustrates sequential stages including peptide identification and optimization, in vitro screening, surface functionalization strategies, preclinical validation, human clinical trials, and regulatory approval. Major barriers to clinical translation include peptide instability, manufacturing costs, regulatory complexity, insufficient long-term clinical evidence, and the potential emergence of microbial adaptation or immune-related adverse responses.

Finally, the field lacks validated outcome measures for the very property that distinguishes this strategy. Whereas antibacterial efficacy can be quantified readily, there are at present no established biomarkers or non-invasive measures that capture the quality of the peri-implant soft-tissue seal—the integrity of the junctional epithelium, the organization of the supracrestal connective tissue, or the resistance of the interface to bacterial ingress. Developing such markers, whether molecular—for example, host-defense peptides and cytokines sampled from peri-implant crevicular fluid—or imaging-based, would allow the pro-sealing effect that this review places at the centre of its argument to be measured directly, in the laboratory and, eventually, in patients. Preliminary work in this direction already exists—the host-defense peptide LL-37, for example, is detectable in peri-implant sulcus fluid, although in at least one study its total amount did not differ between healthy and diseased sites [64]—which underlines both the feasibility of such measurements and the need to develop markers that track the soft-tissue seal specifically. Biomarker-guided monitoring has ample precedent in other clinical settings—systemic inflammatory indices such as the neutrophil-to-lymphocyte and platelet-to-lymphocyte ratios give accessible readouts of inflammatory burden [65], epithelial dual-stain cytology screens for high-risk epithelial lesions [66], and histopathological examination remains central to characterizing tissue change [67]—and analogous, locally sampled molecular or histological markers could be developed for the peri-implant interface. Taken together, these four priorities—controlled clinical trials, standardized head-to-head comparison, long-term durability data, and validated measures of the soft-tissue seal—represent the most consequential next steps for the field (Figure 5).

**Table 3:** Representative quantitative parameters reported for leading antimicrobial and host-defense peptides in titanium dental- and orthopaedic-implant studies: antibacterial activity, anti-biofilm effect, cytotoxicity and host selectivity, surface loading and release, and in vivo evidence. The heterogeneity of assays, target organisms, and reported units across studies is itself a barrier to direct head-to-head comparison and underlines the need for standardised benchmarking (Section 9.7). MIC, minimum inhibitory concentration; MBC, minimum bactericidal concentration; NR, not reported.

Peptide (Origin)	Representative Antibacterial Activity	Anti-Biofilm Effect	Cytotoxicity/Host Selectivity	Loading and Release on Titanium	In Vivo Evidence
**LL-37** (37 aa; human cathelicidin)	Broad-spectrum but salt-sensitive; immobilised on Ti reduces *P. gingivalis* and *S. mutans* [44]	Limits early biofilm formation on coated Ti [44]	Narrowest therapeutic window of this group; host-cell toxicity at higher concentrations (Section 9.2)	Covalently tethered; non-releasing [44]	Ti proof-of-concept: pathogen suppression with a junctional-epithelium-like seal [44]
**KR-12/KR-12-3** (12 aa; LL-37 fragment)	KR-12-3 MIC 156.25 µg/mL, MBC 312.5 µg/mL vs. *S. gordonii* [33]	Inhibits *S. gordonii* biofilm formation [33]	Low; markedly more host-compatible than LL-37; anti-inflammatory (lowers IL-6 and IL-8) [33]	NR	Not yet demonstrated [33]
**GL13K** (13 aa; from salivary protein BPIFA2)	MIC 8 µg/mL vs. planktonic *P. aeruginosa* [68]; 100% kill of *S. gordonii* at 100 µg/mL [69]; L-isomer inactive against *P. gingivalis* [70]	≈3-log reduction in P. aeruginosa biofilm at 100 µg/mL; coating prevents *S. gordonii* biofilm [69]	<10% haemolysis at 1 mg/mL; cytocompatible with fibroblasts and osteoblasts [59,69]	Covalent coating; hydrolytically and mechanically stable with minimal release [37,69]	Osseointegration comparable to that of uncoated controls [25]
**HHC-36** (9 aa; KRWWKWWRR; computationally designed)	Broad-spectrum incl. MRSA and *P. aeruginosa*; ≈99.9% kill of *S. aureus* on coated Ti; >95% reduction [39]	Reduces surface colonisation and biofilm on coated Ti [39]	Minimal cytotoxic concentration ≈200 µg/mL [71]	≈34.7 µg/cm^2^ on calcium-phosphate-coated Ti; burst then sustained release over ≈7 days [71]	Rabbit bone-defect model; supports bone growth [39,71]
**Tet213** (10 aa; KRWWKWWRRC)	≈6-log reduction in S. aureus and *P. aeruginosa* within 30 min on coated Ti [72]	Rapid bactericidal action on coated Ti [72]	Minimal cytotoxic concentration ≈50 µg/mL (more cytotoxic than HHC-36) [71]	Up to ≈9 µg/cm^2^ on calcium-phosphate coating [72]	NR (in vitro Ti to date) [72]

### 9.8. Limitations of This Review

Several limitations qualify the synthesis presented here. The first is intrinsic to its central premise. The bridge drawn between cutaneous wound healing and the peri-implant soft-tissue seal is a conceptual framework, supported chiefly by indirect evidence from dermatology and oral-mucosal biology rather than by direct peri-implant studies; with the partial exception of the single LL-37 experiment discussed in Section 7, the host-defense activity invoked at the peri-implant interface is inferred rather than demonstrated. The reader should therefore distinguish what has been experimentally shown—that certain peptide coatings are antibacterial, cytocompatible, and compatible with osseointegration in vitro and in animal models—from what remains theoretical, namely that these same peptides reconstitute a durable epithelial seal in the human peri-implant environment.

A second limitation concerns the nature and balance of the evidence. Almost all of the work reviewed derives from in vitro assays and small-animal models, and the step from these systems to human peri-implant tissues—with their particular immune milieu, mechanical loading, and polymicrobial challenge—is considerable and as yet untested; the trials needed to close this gap will have to adopt peri-implant disease, rather than a surrogate such as in vitro bacterial reduction, as their endpoint, with adequate follow-up, appropriate comparators, and sufficient power. The literature is also weighted toward positive findings: favourable antibacterial and osseointegration outcomes are reported far more often than failures, whereas cytotoxicity toward host cells, loss of coating activity, and inconsistent antibacterial performance are comparatively under-reported [60,73]. A systematic review of antibacterial surface treatments for dental implants indeed found that most produced some degree of cytotoxicity and that the heterogeneity of peptides, coating methods, assays, and cell types precluded any quantitative synthesis [73]—the same heterogeneity that prevents the meaningful head-to-head ranking of peptide platforms called for in Section 9.7. Within the animal literature itself, however, the antibacterial signal is consistent: a meta-analysis of in vivo studies confirmed a robust advantage for AMP-coated metallic implants over uncoated controls (standardised mean difference −1.74; 95% CI −2.26 to −1.26; *p* < 0.00001), even as it cautioned that between-study heterogeneity was high and that the evidence remains confined to animal models [74].

Finally, this is a narrative rather than a systematic review. Although the search strategy and selection criteria are stated in Section 1, studies were chosen for their relevance to the argument rather than by exhaustive, protocol-driven screening, and no formal risk-of-bias appraisal or PRISMA-based selection was applied. The synthesis is consequently interpretive and subject to selection bias, and is intended to generate hypotheses rather than to deliver the definitive, quantitative appraisal that a future systematic review or meta-analysis should provide.

## 10. Conclusions

Peri-implantitis remains one of the principal threats to the long-term success of dental implants, and the shortcomings of current therapy—the difficulty of decontaminating a structured biofilm on a surface designed for bone anchorage, and the blunt, resistance-promoting nature of antibiotic adjuncts—have created a clear need for preventive strategies that act at the implant surface itself. Antimicrobial peptides are well suited to this role, not only because they kill a broad range of organisms through membrane disruption with a low propensity to select for resistance, but because, as host-defense peptides, they also shape the host response, modulating inflammation, recruiting and activating immune cells, and promoting epithelial and connective-tissue repair.

This review has argued that the second of these properties is what makes the peptide approach distinctive, and that its logic is most clearly understood through the host-defense biology of the skin and oral mucosa. The cathelicidin LL-37 and the β-defensins are central to both the defense and the repair of cutaneous and oral epithelia, and the peri-implant transmucosal interface—a healing epithelial barrier confronting a persistent microbial challenge—is exactly the kind of site where their dual action is valuable. Having established this biological rationale, we now turn from why such peptides are suited to the peri-implant interface to how they can be marshalled, in practice, to protect and reinforce the soft-tissue seal.

The preclinical evidence is encouraging on both counts. Peptide-functionalized titanium preserves and can even enhance osseointegration, partly by tilting the osteoimmune balance toward bone formation, and the recent demonstration that LL-37 immobilized on titanium simultaneously suppresses peri-implant pathogens and builds a junctional-epithelium-like soft-tissue seal offers a direct proof of concept for the dermatological logic advanced here. Significant obstacles remain—peptide stability, the therapeutic window between antibacterial efficacy and host-cell toxicity, manufacturing cost, regulatory complexity, and above all the near-total absence of human clinical data—and these must be resolved before the approach can enter practice.

The direction, however, is clear. Rational, computationally guided design and multifunctional constructs that integrate antibacterial, pro-sealing, and osteogenic activity point toward a new generation of biologically intelligent implant surfaces: coatings that do not merely resist infection but actively help to build and maintain the tissue seal that protects the implant. Because peri-implant disease initiates at the transmucosal region, the surface on which this pro-sealing activity matters most is the abutment or transmucosal collar rather than the endosseous body, and the same host-defense-peptide logic extends beyond titanium to the zirconia and hybrid components now in clinical use (Section 7.4). Bringing the accumulated knowledge of host-defense peptides from dermatology and mucosal biology to bear on this problem is, we suggest, among the most promising routes toward that goal. AMP-functionalized implant surfaces represent one of the most biologically plausible strategies currently under development for the prevention of peri-implantitis, because they simultaneously address the two fundamental drivers of implant success: microbial control and soft-tissue integration. By framing the problem at the intersection of dermatology, oral biology, and biomaterials engineering, we hope to make this application legible to investigators in each of these fields and to invite the cross-disciplinary work that its translation will require. We emphasise, however, that the evidence assembled here derives overwhelmingly from in vitro and animal studies and does not, at present, demonstrate clinical prevention of peri-implantitis; the framework proposed should accordingly be regarded as hypothesis-generating, to be confirmed by standardised large-animal studies and well-designed human clinical trials.

## Figures and Tables

**Table 1:** Representative antimicrobial and bioactive peptides investigated for dental-implant surface functionalization, with their immobilization or delivery strategy, principal microbial targets, experimental models, and main reported outcomes.

Peptide (Origin)	Surface Strategy	Principal Targets	Model	Key Outcome	Ref.
GL13K (human salivary protein)	Covalent silanization; chemoselective Cys-grafting	*P. gingivalis*, *S. gordonii*, *S. mutans*	In vitro; in vivo (rabbit)	Stable, durable coating; antibacterial without impairing osseointegration; pro-osteogenic and anti-osteoclastic (osteoimmune) effects	[25,37,42,43]
HHC-36 (synthetic)	Controlled release from diselenide-bridged mesoporous silica nanoparticles	*S. aureus*, *E. coli*, *P. aeruginosa*, MRSA	In vitro; in vivo (rabbit)	Sustained release ~30 days; >95% antibacterial activity; modulates inflammation and supports osseointegration	[39]
Tet213 (synthetic, broad-spectrum)	Layer-by-layer assembly on a collagen scaffold	Peri-implant pathogens; *S. aureus*	In vitro	Multilayer coating reduces bacterial growth and inhibits biofilm; biocompatibility and immunotoxicity assessed	[38]
KR-12/KR-12-3 (LL-37 fragment)	Cathelicidin-derived peptide (coating candidate)	*S. gordonii*	In vitro	Combined antibacterial and anti-inflammatory activity with host-cell biocompatibility	[33]
LL-37 (human cathelicidin)	Immobilized on nanostructured Ti via polydopamine (LL-37-PD@NT)	*P. gingivalis*, *S. mutans*	In vitro (hGFs); in vivo (rat)	Sustained release >1 week; promotes gingival-fibroblast adhesion, proliferation and ECM synthesis while killing pathogens; forms a junctional-epithelium-like soft-tissue seal	[44]
Bifunctional metal–cell-specific peptides (engineered)	Phage-display Ti-binding sequence + laminin-5/E-cadherin cell-adhesion motifs	Host-cell adhesion (non-antibacterial)	In vitro; in vivo (rat)	Stabilizes epithelial adhesion to the transgingival surface and arrests apical epithelial migration, reinforcing the soft-tissue seal	[45]

ECM, extracellular matrix; hGFs, human gingival fibroblasts; MRSA, methicillin-resistant *Staphylococcus aureus*.

**Table 2:** Comparative profile of four leading antimicrobial and host-defense peptides for peri-implant applications: microbial origin, antibacterial activity, immunomodulatory effect, contribution to the soft-tissue seal, and availability of in vivo evidence.

Peptide	Origin	Antibacterial Activity	Immunomodulatory Effect	Effect on Soft-Tissue Seal	In Vivo Evidence
LL-37	Human cathelicidin (the sole human cathelicidin)	Broad-spectrum; on titanium kills *P. gingivalis* and *S. mutans* [44]	Strong: chemotaxis, cytokine modulation, pro-angiogenic and pro-repair effects [16,17,18,21]	Promotes gingival-fibroblast adhesion, proliferation and ECM synthesis; forms a junctional-epithelium-like seal [44]	Yes—rat immediate-implant model; tight seal resisting tracer penetration [44]
GL13K	Derived from a human salivary protein (parotid secretory protein) [25,37]	Yes: *P. gingivalis*, *S. gordonii*, *S. mutans* (covalent coating) [25,37]	Yes: pro-regenerative immune microenvironment; anti-osteoclastic [42,43]	Not directly demonstrated; cytocompatible with soft-tissue cells	Yes—rabbit femur; osseointegration comparable to controls [25]
KR-12/KR-12-3	Synthetic fragment of LL-37 [33]	Yes: e.g., *S. gordonii* [33]	Yes: anti-inflammatory activity [33]	Not yet studied directly; host-cell biocompatible	Not yet—in vitro evidence to date [33]
HHC-36	Synthetic, rationally optimized peptide [39]	Broad: *S. aureus*, *E. coli*, *P. aeruginosa*, MRSA (>95%) [39]	Yes: modulates inflammation and the macrophage response [39]	Not specifically demonstrated	Yes—rabbit bone-defect model; supports osseointegration [39]

ECM, extracellular matrix; MRSA, methicillin-resistant *Staphylococcus aureus*.

## Data Availability

No new data were created or analyzed in this study. Data sharing is not applicable to this article.
